# A phase I study of imatinib, dacarbazine, and capecitabine in advanced endocrine cancers

**DOI:** 10.1186/1471-2407-14-561

**Published:** 2014-08-02

**Authors:** Daniel M Halperin, Alexandria T Phan, Ana O Hoff, Marie Aaron, James C Yao, Paulo M Hoff

**Affiliations:** Department of Gastrointestinal Medical Oncology, The University of Texas M. D. Anderson Cancer Center, Houston, Texas USA; Division of Hematology/Oncology, Department of Medicine, The Methodist Hospital, Houston, Texas USA; Endocrine Neoplasia Unit, Instituto do Cancer do Estado de São Paulo Faculdade de Medicina da Universidade de São Paulo, São Paulo, Brazil; Instituto do Cancer do Estado de São Paulo Faculdade de Medicina da Universidade de São Paulo, São Paulo, Brazil

## Abstract

**Background:**

Patients with advanced endocrine cancers, such as adrenocortical carcinoma and medullary thyroid carcinoma, have few well-validated therapeutic options. Pre-clinical studies have suggested potential activity of imatinib in these tumors. We therefore sought to establish a safe, novel treatment regimen combining imatinib with cytotoxic chemotherapy for future study in endocrine cancers.

**Methods:**

A standard 3 + 3 dose-escalation design was used with a 21-day cycle, including imatinib on days 1–21, dacarbazine on days 1–3, and capecitabine on days 1–14.

**Results:**

Twenty patients were treated. The most frequent toxicities were edema and fatigue, with dose-limiting fatigue and dyspnea. The recommended phase II regimen is dacarbazine 250 mg/m2 daily on day 1–3, capecitabine 500 mg/m2 twice daily on days 1–14, and imatinib 300 mg daily on days 1–21 of a 21-day cycle. Interestingly, responses were seen in patients with adrenocortical carcinoma, with 1 of 6 patients experiencing a partial response and a second experiencing a minor response, with progression-free survival of 8.8 and 6.4 months, respectively.

**Conclusions:**

The regimen of imatinib, dacarbazine, and capecitabine is well-tolerated. It may have some activity in adrenocortical carcinoma, and further study of this combination or its components may be beneficial for this disease with limited treatment options.

**Trial registration:**

ClinicalTrials.gov identifier NCT00354523, registered July 18, 2006.

## Background

Endocrine cancers are a heterogeneous group of malignancies. Adrenocortical carcinoma (ACC) and medullary thyroid carcinoma (MTC) are challenging cancers to treat if metastatic or unresectable, and few chemotherapy regimens have proven effective for advanced disease.

Medullary thyroid carcinoma (MTC) is a rare tumor arising from the parafollicular C cells of the thyroid gland [1]. Approximately 75% of these tumors are sporadic and 25% are hereditary, associated with the multiple endocrine neoplasia type 2 syndrome (MEN2) [2]. Multiple endocrine neoplasia type 2 is an autosomal dominant syndrome caused by germline activating mutations of the *RET* proto-oncogene which encodes for RET, a receptor tyrosine kinase that modulates C cell proliferation and apoptosis [3–5]. Patients with sporadic MTC do not carry germline *RET* mutations, but 40% of their tumors carry a somatic *RET* mutation, most commonly involving exon 16, conferring a more aggressive phenotype [6–9]. *In vitro* and *in vivo* studies of the most common germline and somatic RET mutations have established their role in oncogenesis [10–13]. *In vitro* studies using a MTC cell line with a *RET* codon 634 mutation demonstrated growth inhibition with imatinib, offering some hope that the drug may have efficacy in this tumor [14].

Systemic cytotoxic chemotherapy for advanced MTC has shown limited tumor response efficacy. Small trials studying dacarbazine, 5-fluorouracil, and doxorubicin [15–20], used alone or in combination, have demonstrated partial biochemical and tumor responses in 10-20% of patients. More recently, inhibitors of the RET kinase, such as vandetanib [21] and cabozantinib [22], have shown evidence of significant progression- free survival benefit, and hence are FDA-approved for the treatment of patients with advanced MTC.

Adrenocortical carcinoma (ACC) is another rare malignancy of neuroectodermal origin with limited therapeutic options. It has an annual incidence of 1–2 cases per million population [23, 24], and a median overall survival that decreases dramatically as a function of clinical stage, ranging from over 10 years for stage I disease to less than 6 months with advanced stage [24]. Most cases are sporadic, but associations have been demonstrated with Li Fraumeni syndrome, Beckwith-Wiedemann syndrome, and MEN 1 [25, 26]. While mitotane has been the mainstay of therapy since it was demonstrated to reduce serum and urine steroid concentrations in over 70% of patients in 1966 [27], high-quality clinical evidence for a survival benefit with any therapy was absent until a recent trial demonstrated the utility of etoposide, doxorubicin, cisplatin, and mitotane (EDP-mitotane) [28]. Molecularly targeted therapies have been of interest [29–31], but none have yet proven successful. Of particular interest was a study demonstrating that adrenocortical carcinomas express cKit and/or the PDGF receptor at some frequency, but are unresponsive to single-agent imatinib [32].

Therefore, MTC and ACC require more effective therapy. As most MTCs have upregulated RET activity and pre-clinical studies using imatinib inhibit MTC cell proliferation and induce apoptosis, this drug has been appealing for treating this disease. ACC could also theoretically respond to imatinib, perhaps when combined with additional chemotherapy to allow for cytotoxicity. We therefore undertook a phase I dose-escalation trial of the combination of imatinib, dacarbazine, and capecitabine in advanced endocrine tumors, including predominantly patients with MTC and ACC.

## Methods

### Inclusion criteria

Men and women of all ethnic groups were eligible if they were > 16 years old with an ECOG performance status of 0–2 and any proven solid tumor for which no curative or standard treatment was available, regardless of prior therapy. Patients needed laboratory evidence of adequate hepatic, renal, and bone marrow function, as well as a negative pregnancy test (if applicable) and an agreement to use barrier contraception throughout therapy.

### Exclusion criteria

Patients were ineligible if they had received chemotherapy or surgery within the last 3 weeks, or radiation within the last 4 weeks. Patients could not have received prior treatment with investigational agents within 28 days of study entry. Severe concurrent illness or ongoing pregnancy or lactation resulted in exclusion, as well. Patients with any other malignancy, except non-melanoma skin cancer or an MEN2-associated cancer, within the prior 5 years were also ineligible. Finally, patients could not be receiving warfarin during the study, though heparin products were allowed.

### Design

All patients provided written informed consent meeting M.D. Anderson Cancer Center Institutional Review Board (IRB) and NCI standards.

The study was designed as a single-arm, open-label dose-escalation study of imatinib, dacarbazine, and capecitabine. Imatinib was given orally on days 1–21, dacarbazine was given intravenously over 1 hour on Days 1–3, and capecitabine was given orally twice daily on days 1–14. A cycle of treatment was defined as 21 days with the next cycle starting on Day 22. A standard 3 + 3 dose-escalation scheme was utilized (Table 1).

**Table 1:** **Dose levels**

Level	Imatinib	Dacarbazine	Capecitabine	Patients (N)	Dose reductions (N)	DLTs ^1^ (N and type)
**−1**	300 mg	250 mg/m2	500 mg/m2 BID	6	0	1 (fatigue)
**1**	400 mg	250 mg/m2	500 mg/m2 BID	6	2	9 (Dyspnea, Fatigue, Diarrhea, Dehydration, Nausea, Ocular Surface Disease, Insomnia)
**2**	400 mg	330 mg/m2	750 mg/m2 BID	6	1	2 (Hypokalemia, platelets)
**3**	400 mg	330 mg/m2	1000 mg/m2 BID	0	0	
**4**	600 mg	330 mg/m2	1000 mg/m2 BID	0	0	
**5**	800 mg	330 mg/m2	1000 mg/m2 BID	0	0	

Dose levels specified for protocol therapy. 1: DLTs – dose-limiting toxicities.

The objective of the trial was to determine the maximum tolerated dose (MTD) of the combination of imatinib, dacarbazine, and capecitabine. Toxicities were graded according to the Cancer Therapy Evaluation Program Common Toxicity Criteria, version 3.0. MTD was defined as the dose level below that producing dose-limiting toxicity (DLT; i.e. any Grade 4 hematologic toxicity and /or non-hematologic toxicity ≥ Grade 3 except alopecia within the first 28 days) in ≥ 33% of patients.

Baseline cross-sectional imaging by computed tomography or magnetic resonance imaging was performed within 28 prior to study enrollment, and response to treatment was measured using the modified RECIST criteria with radiological evaluation every 9 weeks.

Treatment continued until occurrence of disease progression, unacceptable toxicity, or the patient elected to discontinue study participation.

The study was conducted in concordance with the Declaration of Helsinki and approved by the MD Anderson Cancer Center Institutional Review Board as protocol 2004–0475.

### Statistical considerations

Median progression-free survival (PFS) and overall survival (OS) were calculated by the Kaplan-Meier method. Follow-up was calculated from date of study enrollment until date of last contact. All statistical analyses were performed using SPSS version 21.0.

## Results

### Patient demographics

From January 2005 through September 2006, 21 patients were screened and 20 patients were enrolled. Their median age was 52 years (range 33–77 years) (Table 2). Two patients did not complete the first cycle of protocol therapy due to symptomatic or progressive disease, and were excluded from analysis for DLT. The patient population was enriched for patients with MTC and ACC, but also included patient with pancreatic neuroendocrine tumors, melanoma, and transitional cell carcinoma. All patients were metastatic at time of therapy initiation.

**Table 2:** **Baseline patient characteristics**

	Number (%)
**Age (Median)**	52
**Gender**	
**Male**	12 (60)
**Diagnosis**	
**MTC** ^**1**^	7 (35)
**ACC** ^**2**^	5 (25)
**NET** ^**3**^	3 (25)
**Melanoma**	1 (5)
**TCC** ^**4**^	1 (5)
**Prior therapy**	19 (95)
**Surgery**	16 (80)
**Radiation**	10 (50)
**Chemotherapy**	16 (80)
**Prior lines of chemotherapy**	
**0**	4 (20)
**1**	5 (25)
**2**	4 (20)
**>2**	7 (35)

Characteristics of patients enrolled on the study. Total n = 20. ^1^MTC – medullary thyroid carcinoma; ^2^ACC – adrenocortical carcinoma; ^3^NET – neuroendocrine tumor; ^4^Transitional cell carcinoma.

### Dose escalation and maximum tolerated dose

An initial cohort of three patients was enrolled at dose level 1 (dacarbazine 250 mg/m2, capecitabine 1000 mg/m2 twice daily, and imatinib 400 mg) without observing a DLT. The next cohort of three patients was treated at dose level 2, with one patient experiencing grade 3 hypokalemia. An additional three patients were enrolled at this dose level, with one experiencing grade 3 thrombocytopenia. Therefore, three additional patients were enrolled at dose level 1, with all patients experiencing a grade 3 toxicity, including 2 patients with dyspnea and 2 with fatigue. When the next cohort of three patients was enrolled in dose level −1, a single patient experienced grade 3 fatigue. The final cohort of three patients enrolled in dose level −1 and experienced no DLT.

### Safety

The most common toxicities for all cycles were fatigue and edema, each occurring in 65% of patients (Table 3). Edema was mostly grade 1, but 25% of patients described grade 3 fatigue during treatment. The most common grade 3 adverse event was dyspnea, with 30% of patients describing that symptom. Most treatment-related adverse effects were transient, and only one patient required dose reduction.

**Table 3:** **Adverse events**

Toxicity	G1	G2	G3	G4	Total
**Allergic rhinitis**	2	1	0	0	3
**Alopecia**	2	0	0	0	2
**ALT** ^**1**^	1	0	1	0	2
**Anorexia**	2	4	0	0	6
**AST** ^**2**^	1	0	0	0	1
**Bilirubin**	1	0	0	0	1
**Cardiac ischemia**	0	0	1	0	1
**Chest tightness**	1	0	0	0	1
**Constipation**	4	4	0	0	8
**Dehydration**	0	0	1	0	1
**Diarrhea**	5	2	1	0	8
**Distension**	0	0	1	0	1
**Dizziness**	2	0	0	0	2
**Dry mouth**	1	0	0	0	1
**Dry skin**	1	0	0	0	1
**Dysphagia**	1	0	0	0	1
**Dyspnea**	2	2	6	0	10
**Edema**	12	1	0	0	13
**Fatigue**	5	3	5	0	13
**Fever (no neutropenia)**	1	0	0	0	1
**Flushing**	1	0	0	0	1
**Hand-foot syndrome**	1	0	0	0	1
**Hemoglobin**	1	2	0	0	3
**Hypocalcemia**	1	0	0	0	1
**Hypokalemia**	2	0	2	0	4
**Hypomagnesemia**	1	0	0	0	1
**Insomnia**	2	5	1	0	8
**Mood alteration**	0	1	0	0	1
**Mucositis**	4	0	0	0	4
**Nausea**	4	6	1	0	11
**Neuropathy (sensory)**	3	2	0	0	5
**Neutrophils**	0	2	1	0	3
**Ocular surface disease**	1	1	1	0	3
**Ocular/visual**	2	0	0	0	2
**Pain**	12	6	1	0	19
**Palpitations**	1	0	0	0	1
**Platelets**	1	0	0	0	1
**Pleural effusion**	1	0	0	0	1
**Pruritis**	1	0	0	0	1
**Rash**	6	0	0	0	6
**Rigors/chills**	2	0	0	0	2
**Sinus tachycardia**	1	0	0	0	1
**Somnolence**	3	0	0	0	3
**Sweating**	1	0	0	0	1
**Taste alteration**	5	4	0	0	9
**Upper respiratory infection**	1	0	0	0	1
**Voice change**	2	0	0	0	2
**Vomiting**	2	2	0	0	4
**Watery eye**	1	0	0	0	1

Adverse events reported according to the Cancer Therapy Evaluation Program Common Toxicity Criteria, version 3.0. 1: ALT – alanine aminotransferase elevation; 2: AST – aspartate aminotransferase elevation.

### Tumor responses

Overall, 18 of 20 patients reached first restaging. The remaining two had expired from progressive disease. Of those 18 patients, 12 had progressive disease, 6 had stable disease, 1 had a minor response, and 1 had a confirmed partial response as best response to protocol therapy. Intriguingly, both of the responses were seen in patients with ACC, despite both of these patients being previously treated with standard therapy. The remaining 5 patients with ACC experienced progressive disease. No responses were seen in those patients with MTC, but 4 of 5 patients experienced stable disease (Table 4). However, all 4 patients entered the study with stable disease. With a median follow-up of 82 months, the median PFS was 2.3 months (95% CI 2–2.7), with median OS of 18.6 months (95% CI 8.8-28.4). Given the heterogeneity of the patient population, patient-level survival information is given in Table 4.

**Table 4:** **Patient outcomes**

Patient	Diagnosis	PD at entry	Prior chemo	Prior surgery	Prior XRT	Chemo lines (n)	PFS (months)	OS (months)	Best protocol response
**1**	NET^1^	1	1	0	0	1	2.1	13.5	PD
**2**	NET	1	0	0	0	0	0.8	2.3	PD
**3**	MTC^2^	0	1	1	1	4	14.2	93.3	SD
**4**	MTC	0	1	1	1	4	2.3	93.3	SD
**5**	MTC	0	1	1	1	2	7.4	66.7	SD
**6**	MTC	0	1	1	1	3	0.5	4.1	PD
**7**	MTC	1	1	1	1	1	2.3	20.3	PD
**8**	MTC	1	1	1	1	3	2.2	7.1	PD
**9**	NET	1	1	0	1	3	0.5	0.5	Death
**10**	ACC^3^	1	1	1	0	1	6.4	17.5	MR
**11**	MTC	0	0	1	1	0	7.7	88.6	PD
**12**	ACC	1	1	1	1	5	2.4	2.4	Death
**13**	TCC^4^	1	1	1	0	6	2.6	9.1	PD
**14**	ACC	1	1	1	0	2	8.8	39.5	PR
**15**	MTC	0	0	1	0	0	6.3	82.0	SD
**16**	ACC	1	1	1	0	2	1.6	80.7	PD
**17**	ACC	1	0	1	0	0	1.7	79.5	PD
**18**	Melanoma	1	1	1	1	1	2.1	7.8	PD
**19**	ACC	1	1	1	0	2	2.1	13.4	PD
**20**	ACC	1	1	0	0	1	2.0	18.6	PD

Patient-specific characteristics and outcomes. ^1^NET – neuroendocrine tumor; ^2^MTC – medullary thyroid carcinoma; ^3^ACC – adrenocortical carcinoma; ^4^TCC – Transitional cell carcinoma. PD – progressive disease; SD – stable disease; MR – minor response; PR – partial response.

XRT – radiation therapy.; Chemo lines – number of prior chemotherapies.

## Discussion

In this phase I study, we have evaluated the safety of the combination of dacarbazine, capecitabine, and imatinib in metastatic endocrine cancers. The recommended dose regimen for a phase II trial is dacarbazine 250 mg/m2 daily on day 1–3, capecitabine 500 mg/m2 twice daily on days 1–14, and imatinib 300 mg daily on days 1–21 of a 21-day cycle. Dose-limiting toxicities most frequently included fatigue, dyspnea, and minor electrolyte and blood count abnormalities. The combination was otherwise tolerated well.

We also revealed evidence of activity of this regimen in ACC, even in the context of pretreated, refractory disease, a situation for which there are very limited effective therapies. Impact on overall survival is challenging to assess in the setting of a heterogeneous and uncontrolled patient population with respect to previous treatments, however, and overall survival was no better among the two responders than among the five non-responders.

Since this trial was initiated, multiple studies have investigated the *in vitro* and *in vivo* activity of imatinib-based regimens in MTC. Early studies of the *in vitro* effects demonstrated RET inhibition and death of oncogene-addicted MTC cells [14, 33], but these studies demonstrated successful RET inhibition only at serum concentrations that could not be achieved with tolerable doses of imatinib, and subsequent clinical trials of imatinib monotherapy revealed no responses in MTC [32, 34]. In one of these trials of imatinib monotherapy, patients with ACC were included as well, without evidence of clinical response [32]. Additional investigation of this agent in ACC, alone or in combination with cytotoxic chemotherapy has otherwise been lacking, making our combination entirely novel.

## Conclusion

We present here the results of phase I trial of a combination of targeted therapy using imatinib with cytotoxic chemotherapy using capecitabine and dacarbazine in patients with advanced endocrine malignancies. Responses were rare, but occurred exclusively in patients with ACC, a cancer with limited effective therapies. These data should prompt consideration of a phase II trial of such a combination in this disease, given the paucity of other options. Alternatively, our hope is that these results will promote a deeper understanding of the disease biology in those patients who responded, allowing for the insightful and rational development of future targeted therapies.
